# Elements of the Design and Implementation of Interventions to Prevent Violence against Women and Girls Associated with Success: Reflections from the What Works to Prevent Violence against Women and Girls? Global Programme

**DOI:** 10.3390/ijerph182212129

**Published:** 2021-11-19

**Authors:** Rachel Jewkes, Samantha Willan, Lori Heise, Laura Washington, Nwabisa Shai, Alice Kerr-Wilson, Andrew Gibbs, Erin Stern, Nicola Christofides

**Affiliations:** 1 Office of the Executive Scientist, South African Medical Research Council, Pretoria 0001, South Africa; 2 Gender & Health Research Unit, South African Medical Research Council, Pretoria 0001, South Africa; samantha.willan@mrc.ac.za (S.W.); Nwabisa.Shai@mrc.ac.za (N.S.); Andrew.Gibbs@mrc.ac.za (A.G.); 3 School of Public Health, Faculty of Health Sciences, University of the Witwatersrand, Johannesburg 2193, South Africa; Nicola.Christofides@wits.ac.za; 4 John Hopkins Bloomberg School of Public Health, John Hopkins University, Baltimore, MA 21211, USA; lheise1@jhu.edu; 5 School of Nursing, John Hopkins University, Baltimore, MA 21211, USA; 6 Project Empower, Diakonia Centre, 20 Diakonia Ave, Durban 4001, South Africa; laura@projectempower.org.za; 7 Social Development Direct, Finsgate, 5-7 Cranwood Street, London EC1V 9LH, UK; alice@sddirect.org.uk; 8 Gender Violence and Health Centre, London School of Hygiene and Tropical Medicine, Keppel Street, London WC1E 7HT, UK; erin.a.stern@gmail.com

**Keywords:** intimate partner violence, gender-based violence, prevention, intervention, programme, design, implementation, gender, women’s health

## Abstract

Intimate partner violence (IPV) has a large and sustained impact on women’s mental health, and so effective prevention is critical. A review of 96 rigorous evaluations of interventions for their impact on violence against women and girls (mostly IPV) found that several intervention approaches were effective. However, not every evaluation of a ‘successful approach’ showed success in reducing IPV. In order to understand what else impacts success, we analysed practitioners’ accounts and documentation of the design and implementation of seventeen interventions evaluated as part of *What Works to Prevent Violence against Women and Girls (VAWG)*. Six features were identified as characteristics of all successful interventions: a rigorously planned intervention with a robust theory of change (ToC), attuned to the local context; addressing multiple drivers of VAWG; support for survivors; working with women and men; implementing at optimal intensity and having sufficient, well-selected, trained and supported staff and volunteers. Four features were necessary for success when relevant for the intervention approach: gender and social empowerment group activities and promoting positive interpersonal relations; participatory learning methods, emphasising empowerment, critical reflection and communication skills; carefully designed user-friendly manuals systematically followed; and when working with children, having an age-appropriate design with time for learning and an engaging pedagogy. This analysis provides the IPV prevention field with critical information for enhancing the impact of group- and community-based interventions in IPV prevention and through this strengthening women’s mental health.

## 1. Introduction

Intimate partner violence (IPV) and rape are globally pervasive problems, with a particularly high prevalence in low- and middle-income countries [1]. The health impact of IPV and rape have been elucidated over several decades and the findings show that the immediate impact of injuries and mortality is a small part of the burden of ill-health attributable to IPV and rape exposure. The overall impact is seen in the evolving mental health consequences, sexual and reproductive health problems and elevated risk of sexually transmitted diseases, particularly HIV, with cycles of health problems transmitted across generations to children exposed to IPV [2,3,4,5,6]. In the arena of mental health, depression and anxiety, suicidality, self-harm, harmful substance use, post-traumatic stress disorder (PTSD) and personality disorders are more commonly found among women exposed to IPV [4,5,7,8]. The mental health impact is recognised as mediated by responses to chronic stress associated with experiencing IPV, which is most commonly a repeated or enduring occurrence, with emotional and/or economic aspects of violence being the most pervasive forms. Harmful alcohol use and use or misuse of other drugs are also commonly part of pathways, operating both as consequences and drivers of IPV [9]. Prevention of experience of intimate partner violence by women is thus critical for improving women’s mental health.

Over the last decade, prevention of violence against women and girls (VAWG) has become an increasingly important part of development programming. The term VAWG is mostly used to refer to IPV and non-partner rape, but also captures sexual harassment, violence at work, violence at home from other family members, and gendered violence in schools. A comprehensive review of the field conducted by Kerr-Wilson et al. found that by 2019, 95 separate interventions had been subject to rigorous testing for their impact on VAWG outcomes (mostly IPV) [10]. Nine intervention approaches were shown to be effective in reducing the self-reported risk of women or girls experiencing intimate partner violence (IPV), or non-partner rape, when evaluated in two or more randomised controlled trials (RCTs) or quasi-experimental studies with a control arm. Two-thirds of the evaluations were in low- and middle-income countries. There were eight interventions that sought to reduce IPV among women from the general population through reducing harmful alcohol use, and in some cases also strengthening mental health, and six of the nine rigorous evaluations of these interventions showed evidence of positive effect [10]. Furthermore, two interventions evaluated with female sex workers sought to reduce their use of alcohol and drugs as a way of reducing their violence exposure and were shown to be successful in reducing violence experienced from clients [11]. However, with these, as with other approaches, the review found that some intervention evaluations did not show a reduction in IPV, raising the question of what influences intervention impact beyond the intervention approach? Further, are there aspects of the design and implementation of interventions associated with greater success?

These are not easy questions to pursue given that published descriptions of the design and implementation of interventions are often thin. However, the UKAID-funded What Works to Prevent Violence Against Women and Girls? (*What Works*) programme, provided a valuable opportunity to gather comparable information across its 17 interventions (see below) and reflect on which elements of their design and implementation seem to be associated with greater evidence of impact. These interventions were designed to reduce IPV, non-partner sexual violence, workplace violence and peer violence amongst children and were evaluated in studies across 13 countries in Sub-Saharan Africa, and Central and South Asia between 2015 and 2019. The aim of our paper is to reflect on the design and implementation of the interventions in *What Works* and consider whether there are aspects of these that distinguished those that were more effective in preventing and/or reducing VAWG, with a view to guiding future programming. This paper follows an initial report on this subject that has further information on each intervention’s design and implementation [12].

## 2. Materials and Methods

We reviewed the curricula, most of which are available online (http://www.whatworks.co.za/, accessed on 16 November 2021). We developed a framework for documenting the details of the design and implementation of interventions in Excel and pilot tested it. A worksheet captured the intervention content, context and setting, duration, elements, delivery, length per session and period over which the sessions were delivered, ToC, and any implementation deviations. Another captured the number of staff and volunteers in management and on the front line, selection criteria and processes used to identify staff/volunteers, their background, the duration of the initial training and any practice periods, in-service training, and the extent of monitoring and support provided during implementation. A third worksheet captured the number of communities or people reached in different activities.

The Excel sheets were populated by the authors (mostly RJ and SW) in discussion with the lead intervention implementer, and usually others from the project, and compared against the intervention papers, fact checked by the interviewee and any discrepancies discussed. The first author used her knowledge of the interventions to moderate descriptions, if needed, to provide consistency across reports. Table 1 shows the definitions that were applied in this analysis.

Hypotheses about critical factors influencing intervention success were developed from practice-based knowledge of the author team [13] and assertions from the literature on what constitutes good practice in intervention design and implementation, and tested through reviewing from the intervention accounts. The face validity of findings was tested through presentations to different audiences of practitioners, researchers and donors, as well as peer reviewers of the report, with the feedback used to refine it. The views presented here are those of the authors and not necessarily those of our practitioner colleagues. A more detailed account of the interventions is provided in their respective methods, process evaluation or outcome papers, and brief intervention summaries may be found on the What Works website https://whatworks.co.za/resources/policy-briefs (accessed on 16 November 2021).

## 3. Results

### 3.1. Components of Different Interventions and Their Evaluations

Table 2 provides a high-level overview of the 17 interventions, all were multi-component. Seven were mostly based on community activism or included elements of it. Four had economic empowerment components, one being implemented in a workplace. All but two had a formal structure or curriculum and these had elements of skills building in all but two cases. One of the interventions was a psychotherapeutic intervention using the Common Elements Transdiagnostic approach (CETA) and three others included counselling for survivors of violence, or couples. Eleven of the interventions were evaluated through RCTs, two in quasi-experimental studies with a control arm, three had mixed method, non-controlled evaluations and one, a modified interrupted time series. The interventions were in Central Asia (3), South Asia (5) and Sub-Saharan Africa (9). In four of the 11 RCT evaluations, one of two quasi-experimental studies with control arms and three of the four other evaluations, the conclusion was that the intervention had reduced violence as the analysis showed a significant reduction in self-reported IPV experience (women) or perpetration (men) or peer violence when compared to the control arm or else in a trend across 3 or 4 time points.

**Figure 1:**
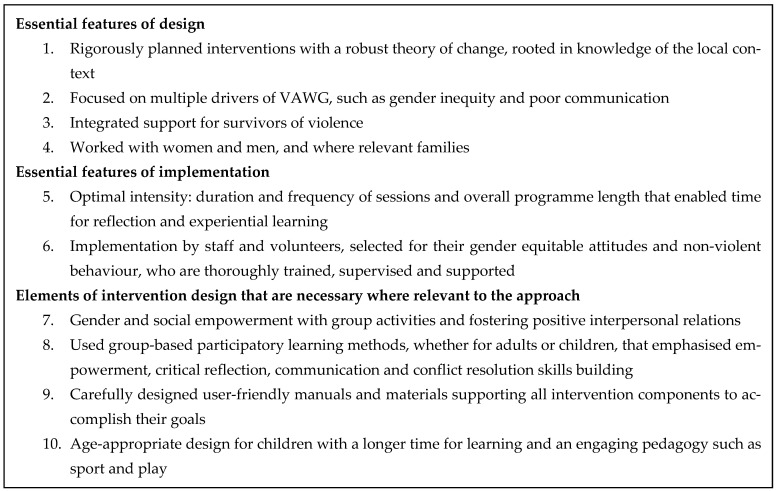
Ten elements of the design and implementation of What Works interventions found to effectively reduce VAWG.

Despite the diversity among the *What Works* interventions and evaluation settings, ten core elements emerged as influencing whether the intervention was able to reduce violence. These largely cut across intervention categories, with some exceptions discussed below, and are shown in Figure 1. Table 3 shows how the ten elements of effective interventions mapped on to the 17 interventions. In the table a solid black dot is given to interventions that robustly included the element. A diamond indicates that an element was present but limited in some way. Where there is no dot, the intervention did not include the element.

Seven of the nine effective interventions included all four essential elements of intervention design and the two essential elements of intervention implementation. The other two effective interventions were interventions working with children to prevent peer violence delivered by Right To Play in Pakistan [14] and the Peace Education in Afghanistan [15]. These showed strong evidence of impact but did not include any support for survivors. We suggest that because peer violence is a different type of violence to IPV and non-partner rape, individual survivor support may not be as essential.

In addition to the key elements, Table 3 also includes additional intervention design elements that were found to be necessary for specific intervention approaches. The additional elements were: (1) a focus on gender and social empowerment and developing positive interpersonal relations through group activities; (2) participatory learning methods, emphasising empowerment, critical reflection and communication skills; (3) having carefully designed user-friendly manuals systematically followed; and, for children’s interventions only; (4) having an age-appropriate design with time for learning and an engaging pedagogy.

Most of the effective interventions working with adults included all additional design elements (bar age appropriate), although there were exceptions. One exception was a couples psychotherapeutic counselling intervention (VATU), which was delivered individually (apart from an unsuccessful attempt to deliver to groups very early on), and so did not have group activities nor participatory learning methods, but did have carefully designed, user-friendly manuals. The other intervention—Rural Response System, Ghana—was based on community activism to change social norms and had a strong gender empowerment focus but did not have group workshop-style activities, but provoked group discussion at social gatherings and it worked with couples directly affected by IPV.

For the two effective or promising interventions working with children there was a similar pattern. In Pakistan, all four key additional elements were present. While the intervention in Afghanistan included all additional elements, apart from the participatory group work. Among the interventions that were not found to reduce VAWG, Table 3 shows that some had some of the necessary elements in their design, of the intervention that were necessary for the approach, but without all the essential elements of design and implementation the intervention was not effective in VAWG prevention.

### 3.2. Ten Core Elements of Intervention Design and Implementation

#### 3.2.1. Design

##### Rigorously Planned Interventions with a Robust Theory of Change, Rooted in Knowledge of the Local Context

Our findings highlight the importance of carefully planned interventions, built on deep local knowledge, with all relevant aspects of the intervention, and designed around a well-conceived ToC. In most cases, considerable thought had been given to ensuring that different parts of the intervention were foundational for change and could achieve their goals. Many of the more successful interventions had been developed over years prior to the *What Works* programme, based on initially on formative research, building on practitioner experience with interventions, had been piloted and their manuals and materials then refined. Additionally, there had been formative research in the new setting. Major weaknesses in the fundamental design and ToC of an intervention proved to be critical, even if there were other areas of strength.

For example, the Women’s Empowerment Programme (WEP) in Afghanistan was not designed as an IPV prevention programme; rather it was hypothesised that the core elements of social and economic empowerment would be sufficient to reduce VAWG. The evaluation showed, however, that without a ToC aimed at VAWG prevention, key elements were missing and the women’s empowerment that was achieved was not sufficient to prevent IPV [16]. There were also key conceptual weaknesses in the HERrespect intervention in Bangladesh [17]. This was delivered to women garment factory workers as the main target group and the intervention design was based on an assumption that women would be economically empowered as they were earning. However, we observed that many women were effectively ‘wage slaves’, with very little control over their earnings, and also were stigmatised as working women [18].

##### Addressing Multiple Drivers

The literature on violence prevention emphasises the importance of multiple component interventions (e.g., [19,20]); however, our findings have clarified that what is critically important is addressing multiple drivers of violence, including the most important drivers in the participants’ lives [21]. This can be done with one component, as in the case of Stepping Stones and the Indashyikirwa couples’ interventions, which both challenged gender inequity and the use of violence, while building stronger, more harmonious relationships, empowered with better communication [22,23]. Our conclusion in respect of multiple drivers is supported by the recent comprehensive review of what works in VAWG prevention [10].

The Sonke Change intervention, in South Africa, encountered problems due to inadequate attention to local drivers of violence in its design [24,25]. It focused almost exclusively on challenging gender inequality and patriarchal privilege, yet it was implemented in an area with multiple drivers of violence, where poverty, substance abuse, poor mental health and poor relationship skills were major issues, and it did not provide survivor support [24]. This probably contributed to the intervention’s inability to deliver the intended results.

##### Support for Survivors

Providing support for survivors was an important part of many of the interventions. Survivor support varied, and in some interventions, also included support for men as survivors of violence or for their mental health generally. VATU in Zambia was psychotherapeutic in nature and delivered to women experiencing severe violence, and their male partner [26]. In another approach, the Indashyikirwa community intervention in Rwanda had survivor safe spaces offering aid to female and male survivors, as well as prevention/empowerment activities primarily targeted to women [27]. In another approach, the Rural Response System (RRS) in Ghana [28] had community activists trained in mediation skills, and they enabled conversations between couples around problems in relationships, as well as conveying anti-VAW messaging.

In South Africa, at the Stepping Stones Creating Futures workshops, men, as well as women, were involved in workshops that providing collective support and an opportunity to express the psychological impact of stressors in their lives. Some interventions had arrangements for referral of survivors who were identified as needing this, but few took referrals and it did not appear to influence intervention success.

##### Work with Women and Men, and Where Relevant, Families

It was very important that women experiencing violence, their husband/ partner and any other potential perpetrators or key influencers, were all engaged in the intervention appropriately. Most of the interventions were developed for both women and men in intimate relationships; however, in Central and South Asia, the socio-cultural context pointed to the importance of work with families. The intervention was first developed in Tajikistan as Zindagii Shoista [29], and later adapted for Nepal as Sammanit Jeevan [30]. These cultural contexts involving other family members, particularly in-laws, was considered critical for enabling young women’s attendance and supporting them to implement new ideas.

There were some interventions that were not successful, at least partly because they were developed in a way that was gender imbalanced. The Sonke Change intervention in South Africa was positioned primarily to work to challenge patriarchy with men. While many women were involved, the programme did not meet women’s needs to discuss and work through their experiences of violence or access counselling or services. The Samvedana intervention in India, conversely, was designed around women sex workers but sought to involve their male intimate partners [31]. The intervention ultimately had a critical weakness as it was not developed from the beginning with and for men, and the design did not sufficiently accommodate the fact that these mostly married men were in covert extra-marital relationships with sex workers and were uncomfortable discussing this in public. Thus, it could not meet the design ambition of working with the woman and her intimate partner.

#### 3.2.2. Implementation

##### Optimal Intensity: Duration and Frequency of Sessions, and Overall Programme Length That Enables Time for Reflection and Experiential Learning

Having an optimal intensity of the programming was important: with sufficient staff or activists deployed; and appropriate programme length; number, duration and frequency of sessions; none of the interventions that had sub-optimal intensity were effective. Interventions using community activists seemed to follow a rule that ‘more is better’. Successful interventions in Ghana and the DRC had a large number of community activists deployed in comparison to the population to be reached, lots of activities and a long duration. For example, in Ghana, there were 5 paid staff and 120 community activists working with a population of 159,869 adults aged 18–60 years living in 20 communities and activities spanned 18 months. In the DRC, there were 225 community activists, 30 gender champions and 75 faith leaders trained and engaged in the community of 15 villages and 216,363 residents (estimated 99,526 adults aged 18–60 years, applying proportions from Ghana), and intervention activities spanned 30 months. The ratio was 1 activist to 1229 adults in Ghana, and 1 activist to 302 adults in DRC. The successful Indashyikirwa couples programme in Rwanda lasted 5 months and had an additional element of community activism to the extent that 25% of 1680 intervention participants undertook activism for an additional 22 months [23]. Interventions with community activists that were unsuccessful all experienced challenges with delivery intensity. The community activism component of Change Starts at Home in Nepal was extremely light touch, with activists having one engagement per month over three months [32]. The Sonke Change intervention only had 6 staff and 18 community activists working at any time in 9 clusters in a large community.

Workshop-based interventions needed to be sufficiently time-intensive, and most successful ones were held weekly, or twice-weekly, enabling sessions to build on each other with time for experiential learning and reflection between sessions. Workshops mostly lasted for two to three hours at a time, enabling in-depth discussions. Most of the successful workshop-based interventions were therefore 40 to 50 h long, generally delivered in 13–20 sessions. Yet, long length did not necessarily ensure success.

A number of interventions revealed the challenges of light-touch intervention delivery. The HERrespect intervention in garment factories was designed as an 18 h curriculum, comprising of 6 sessions, each lasting three hours. The whole intervention was meant to be delivered over 6 weeks. However, the factories would not release the staff for this time and groups were only allowed to meet monthly, leading to delivery over about 9 months, and the sessions at the start of the programme were shortened to 1.5–2 h when levels of support from management were particularly low. With sessions trimmed and too far apart, it was hard for facilitators to build on previous discussions. As discussed more below, the successful interventions with children were delivered for at least 30 min weekly over two years. This contrasted markedly with IMpower and Sources of Strength, which were a total of 12 h long and were unsuccessful in impacting violence [33].

##### Staff and Volunteers: Selected for Their Gender Equitable Attitudes and Non-Violent Behaviour, and Thoroughly Trained, Supervised and Supported

Careful selection, training and support for staff and volunteers was very important for intervention success. With community activism and workshops, the more effective interventions had a very careful selection process or, as in the case in Ghana, nomination from local communities, for activists. This ensured that they had gender equitable and non-violent attitudes and behaviours prior to their training. Several of the more successful interventions used experienced facilitators with proven skills, drawn from the local community. In contrast, the Sonke Change intervention recognised that community activists should themselves be non-violent and gender equitable yet this was not made a requirement on recruitment. They took ‘all-comers’ and training was brief. It is very difficult within typical training periods to change attitudes and behaviors on gender from very conservative to sufficiently equitable to appropriately facilitate gender transformative programming, therefore selecting staff who already have the desired characteristics is key.

The more successful interventions also trained staff for longer and this included staff and volunteers being taken through the entire intervention as participants. For Stepping Stones and Creating Futures, the trained lasted six weeks, with two weeks for attending the intervention as participants, two weeks of other content in the subject matter and how to facilitate, and two weeks practicing the sessions as facilitators. Ongoing support and supervision of staff and volunteers was also a notable feature of successful interventions, and this included psychosocial support. Successful programmes often included weekly discussion sessions among facilitators to jointly problem-solve challenges and provide de-briefing. Where teams were geographically spread out, good practice included regular telephonic contact by team leaders.

Change Starts at Home, in Nepal, had difficulties stemming from its great length (70 h) and having a very large number of staff facilitating the workshops. This made training, support and supervision hard. The 72 staff ran the 72 groups, thus there was no opportunity to improve skills with experience. Further the intervention was developed iteratively and so the staff were not able to gain a full understanding of it, and experience its transformatory power, before leading sessions and it was not tested and refined before it was implemented. With light touch supervision, there was very little opportunity for quality assurance or establishing fidelity to the intervention.

#### 3.2.3. Design Elements Necessary Where Relevant to the Approach

##### Gender and Social Empowerment with Group Activities and Fostering Positive Interpersonal Relations

Most of the interventions (for both adults and children) involved gender and social empowerment and were developed from a perspective that behaviour change is a collective process, rather than one of individual change alone, and requires challenging deeply held values and opinions [34,35,36]. They sought to build an understanding of the gendered nature of violence, to build gender equity, foster conflict resolution and positive interpersonal relations. Exceptions to this included VATU in Zambia, which provided individual psychotherapy to men and women who were couples, and thus fostered positive interpersonal relations in individual sessions but did not have a strong gender and social empowerment component. The Rural Response System intervention in Ghana had gender empowerment and positive interpersonal relations centrally woven through its work and messaging; however, it did not have any group workshop-style activities. The IMpower and Sources of Strength interventions in Kenya did include these elements in the interventions. Their lack of impact also may have stemmed from physical self-defence exercises being hard to teach in very small classrooms and other sessions using didactic rather than participatory teaching, with demonstrations in front of the class and chanting slogans.

##### Using Group-Based Participatory Learning Methods, for Adults or Children, That Emphasise Empowerment, Critical Reflection, Communication and Conflict Resolution Skills Building

Eleven of the interventions were designed with participatory learning methods, often building upon approaches rooted in Freirean philosophy [37,38], used in workshops or other group sessions, and six of these interventions were shown to be effective. It was apparent that participatory learning methods alone were insufficient to make an intervention effective if there were other intervention weaknesses. However, in other circumstances they were critical to the intervention’s success. This has been most effectively explored through qualitative research, which found participatory activities to be central to enabling personal and group transformation through critical reflection and working through problems and positions [39,40].

Two of the interventions that were ineffective only used participatory methods in a very small element of their work. The Sonke Change intervention curriculum predominantly used approaches that engaged at a cognitive level with gender relations, rather than providing space for community members to understand, engage with and re-shape emotions. Similarly, outside of engagement with State actors (as opinion leaders) and the women’s safe spaces, the Indashyikirwa community intervention in Rwanda was delivered chiefly as an awareness raising intervention with volunteers frequently making very short contributions in public meetings supported by posters. Only a small component, delivered mostly in the last few months of the programme, involved more participatory group engagement. This had not been the intention, rather how the programme was implemented.

##### Carefully Designed User-Friendly Manuals and Materials Supporting All Intervention Components

Most of the interventions had well designed user-friendly manuals for training and supporting staff and volunteers. This was an indication of the planning of the intervention and mostly the manuals had content that mapped on to their ToC. For the most part this included a manual that presented a clear curriculum approaches from beginning to end. An exception was the Sonke Change intervention in South Africa that did not take groups of participants through the entire intervention in its workshops, but rather treated its manual as a resource to dip onto for two-day workshops with enrolment not requiring a commitment to attend all workshops. As a result, attendees joined any workshop they wanted to, as such some attendees may have attended just one or two of the workshops, or even the same one twice.

##### Age-Appropriate Design for Children with a Longer Time for Learning and an Engaging Pedagogy Such as Sport and Play

The pedagogical approach of intervening with children is particularly important and learning needs to be empowering, engaging and fun [41]. The two successful interventions with children used participatory, fun, group-based methods, with the goal of empowerment at their core. These were the play-based life skills intervention of Right to Play in Pakistan and Peace Education in Afghanistan. These interventions with children, who were pre- and early-teenage, included critical reflection, communication and conflict resolution skills building, which have all been shown to be valuable elements in interventions with adults, provided through participatory activities and, in the case of Right to Play, structured games [42]. The more effective interventions for children were both delivered consistently over two years, reflecting children’s need time to develop learning skills of critical reflection, problem-solving, communication, and collaboration on a path to non-violent behaviour. Right to Play also notably used a very engaging pedagogy, centred around sport and play. The interventions with children that did not use these approaches were not effective.

## 4. Discussion

### Implications of These Findings for Best Practice in VAWG Prevention

Preventing IPV is critical for removing a major driver of women’s mental ill-health. To our knowledge, this is the first endeavour to look across multiple VAWG prevention interventions and ask the question of why some interventions are more effective than others when using a proven intervention approach. Although previously reflections on good practice have been published [30,42,43]. We have focused our attention on areas of intervention design and implementation associated with success, and have identified ten aspects, among which, six we argue are essential for all interventions and four are required for appropriate interventions. The six essential aspects for effective interventions are: (1) being rigorously planned, with a robust ToC, rooted in knowledge of the local context; (2) having a focus on multiple drivers of VAW; (3) integrating support for survivors; (4) work with women and men, and where relevant families; (5) delivering at optimal intensity, enabling time for reflection and experiential learning; and, (6) implementing through carefully selected and well trained and supported staff and volunteers. Our findings complement those of research that has reflected on how interventions work within individual intervention studies and across groups of studies [39,40,44]. We have used strengths or weaknesses of the design as implemented for our reflection in this paper, rather than strengths or weaknesses of the original design, and acknowledge that for some interventions these differed. The task we have undertaken is not an exact science, and the arguments presented draw on our professional experience of working in the VAWG prevention field and in-depth knowledge of different interventions in the *What Works* portfolio, i.e., our practice-based knowledge [13].

Our findings are important for commissioning and planning VAWG prevention programmes. *What Works* findings showed that VAWG prevention can be achieved within reasonable funding timeframes; however, all the programmes had multi-year funding. Core funding and multi-year funding are essential for implementers to consolidate learnings and build their work. Our observation that programming seeking change in social norms or work with children takes a considerable amount of time, leads us to conclude that such work should be funded for at least three years, including an adequate inception phase. Short-term funding for VAWG prevention has a value in supporting intervention adaptation or implementing shorter programmes; however, it is not useful for funding social norms change work as this is not achieved in a short timeframe.

From planning to implementation, projects need to include and fund careful selection and initial training of staff and volunteers, as well as in-service training, supervision and support. Vicarious trauma must be addressed. Personnel must be gender equitable and non-violent, and so NGOs that are new entrants to the VAWG prevention field may need to employ new personnel for the work. Ideally, they should share characteristics with intervention participants of age, gender, being married or ever partnered, and being from the community. They must be respected by the community in order to relate to and influence participants. After training, they should be asked to demonstrate that they have the appropriate skills before they are deployed.

Our findings show that interventions need to be acutely sensitive to the local context. There is now a good understanding in the field as a whole about drivers of violence [21], but in local settings some will be more important than others. Local information is needed for intervention adaptation and participatory formative research with a desk-based review are key. This requires a sufficiently long inception phase, to enable proper adaptation, testing, staff selection and training, which donors must resource. The *What Works* experience showed that this process took a year for a substantial adaptation, including the contracting projects, formative research, adaptation, as well as hiring and training of staff.

This review has been restricted to the interventions evaluated in *What Works* and we acknowledge that there may be valuable lessons from interventions that are not part of this portfolio and perhaps also from interventions using other strategies than the group, school and community-based strategies of our interventions. We knew whether interventions were effective in VAWG prevention prior to the review and so cannot exclude all bias, although we were conscious that this may have been present and worked hard to reduce it by having multiple authors and reviewers of the work. Although *What Works* standardised some elements of the evaluation such as questionnaires, there were differences in the intervention evaluation designs. We cannot exclude the possibility that our conclusions about the effectiveness of a particular intervention may have been different had the evaluation been designed differently, or if implementation had been somewhat different. However, we have sought in this paper to provide a high-level analysis of intervention design and implementation characteristics, drawing from learnings across several interventions. We note that where interventions were found not to work, we conclude it was due to not all the necessary aspects being included in the intervention. This does not necessarily mean that the particular intervention could not work if there were an adaptation with the identified problems addressed. In cases where interventions were not shown to work, we believe it is unlikely that our conclusions about the interventions would be greatly changed had the research been conducted somewhat differently. We have not always known which elements of an intervention design or implementation made the key difference to its effectiveness, and we have thus had to draw on some of our practice-based knowledge and highlighted possible candidates.

All the What Works interventions and studies were completed prior to the COVID-19 pandemic and we acknowledge that due to restrictions such as social distancing, some of the recommendations (e.g., residential training) may be harder to implement and that others will need to be adapted for virtual spaces rather than face to face. We are not positioned to suggest how to adapt training and implementation to respond to the pandemic and it is not the purpose of this paper to do so.

## 5. Conclusions

Preventing IPV is vital for improving the health of women, and particularly important for improving mental health outcomes. Research has shown that this can be achieved through carefully developed and implemented prevention programming. In this paper, we have offered reflections on the interventions in the *What Works* portfolio and presented structured reflections on aspects of the design and implementation of the interventions to address the question: what intervention features are influencing intervention impact on VAWG? Our conclusions have drawn on the lessons from the six years of *What Works* interventions implemented in three global regions and are also infused with our practice-based knowledge of VAWG prevention across several decades. Previously reflections on intervention effectiveness have focused on the success or otherwise of different approaches to interventions, this to our knowledge is the first attempt at structured reflection on design and implementation with high level conclusions that pertain across different approaches. We have agreed that attention to these matters critically influenced the success of interventions in reducing VAWG. This conclusion is invaluable for guiding future programming.

## Figures and Tables

**Table 1:** Definition of Terms.

Term	Definition
Cash transfer	Money given to a recipient with or without conditions.
Communication skills	Skills include non-verbal and listening skills, demonstrating empathy and expressing thoughts and feelings effectively.
Community	A group of people who share a characteristic that at times unite them and mark them as different from others. A community can be formed around residential geographical location, ethnicity, minority group membership, religious sect, etc.
Community activism	Staff or volunteers trained and deployed to work with fellow community members to change social norms and practices. These approaches usually involve face-to-face conversations with community members, either with door-to-door visits, or public conversations through engagement in public areas, sometimes at an event, drama, mural or film. Community leaders or public officials may be actively engaged, as well as and people or couples experiencing violence. There may be workshops for community members. Sometimes community activism focuses on advocating for change iin policy and practice.
Community leader or service provider outreach and training	Small group or one-to-one discussions with religious or political leaders or service providers (police, justice, health sector, social work), and can include formal training or workshops. Engagements introduce the intervention, often seek changes in working practices and enable more aid for women experiencing VAWG.
Community radio, TV and announcements	Interventions through these media may include public awareness messaging, broadcasting educational drama, discussions, interviews with key people, etc.
Counselling or therapy	A process of meeting with a counsellor/therapist individually or in small groups to work through thoughts, feelings, relationship issues, problematic behaviours or somatic responses.
Couples programme	A programme attended by two people who are in a formal or romantic relationship, either married, cohabiting or dating partners, usually with some sessions focused on relationship strengthening.
Critical reflection	A process of identifying, questioning, and assessing deeply-held assumptions about our knowledge, how and why events and issues are perceived as they are as well as beliefs, feelings, and actions.
Economic empowerment activities	Activities undertaken with the goal of elevating participants’ economic status either through micro-loans or training, support and/or finance assistance given to start small businesses.
Experiential learning	Learning derived from putting new ideas and skills into practice and reflecting on experiences in so doing.
Gender transformative programmes	Policies and programs that seek to challenge rigid gender norms and promote gender equality as part of achieving program objectives.
Intervention	Activities designed and implemented with a specific intention, e.g., prevention of VAWG. Interventions are ideally designed around a ToC.
Intervention component	A discrete aspect of an intervention that has a unified methodology, for example providing cash transfers or a series of workshops.
Life skills programme	Designed to convey generic skills for adaptive and positive behaviour to enable participants to meet challenges in life. Usually these include communication skills, critical reflection, problem-solving, decision-making, collaboration, personal and social responsibility.
Multiple component	An intervention with two or more components, e.g., microloans and gender programming.
Participatory methods	Learning approaches that empower group participants to co-create knowledge, usually through group exercises with critical reflection and discussion of thoughts, feelings, beliefs, insights, etc.
Self-defence skills	Skills to prevent, de-escalate or end an attempted assault, including physical and verbal skills to anticipate and avoid risk
Skills building intervention component	A component of an intervention that is designed to develop skills in communication, conflict management, problem assessment, risk reduction, including very specific skills such as running small businesses or self-defence.
Small business skills	A set of skills to enable the development and implementation of successful small businesses. These usually cover all or some of assessing the market, planning resource needs, budgets, skills requirements, sales and savings.
Theory of change (ToC)	A map for intervention design and evaluation of impact, setting out how an intervention should bring about change. It includes a problem statement, analysis of barriers to change, inputs to overcome barriers, outputs and short-term outcomes on the path to the overall goal.
Workshop or curriculum	Structured programme that is usually designed around a ToC with later sessions building on earlier ones and delivered to a group of participants.

**Table 2:** Overview of the components of the different interventions, type of evaluation and outcome.

	Intervention Component							Evaluation	Outcome
	Community action teams	Community radio or announcements	Working with community leaders or public officials	Economic empowerment: cash transfer or wages, small business or livelihood skills	Workshop or curriculum	Therapy or counselling	Skills building	RCT, quasi-experimental study or modified interrupted time series/mixed methods	Evidence of VAWG or peer violence reduction
Indashyikirwa community activism, Rwanda	●		●			●		RCT + qual	No
Rural Response System, Ghana	●	●	●			●		Quasi-experimental + qual	Yes
Sonke Change, South Africa	●				●		●	RCT + qual	No
Transforming Masculinities, DRC	●		●		●	●	●	Non-controlled, mixed methods + qual	Yes
Change Starts at Home, Nepal	●	●			●		●	RCT + qual	No
Zindagii Shoista, Tajikistan				●	●	●	●	Non-controlled, mixed methods	Yes
HERrespect Bangladesh	❖	●		●	●		●	Quasi-experimental	No
Sammanit Jeevan, Nepal				●	●		●	Non-controlled, mixed methods	Yes
Women’s Empowerment Programme, Afghanistan				●	●		●	RCT + qual	No
Indashyikirwa couples, Rwanda	❖		●		●		●	RCT + qual	Yes
Samvedana Plus, India	❖		●		●			RCT + qual	No
Stepping Stones Creating Futures South Africa				●	●		●	RCT + qual	Yes for men, no for women
Impower, Kenya					●		●	RCT + qual	No
VATU, Zambia					●	●	●	RCT + qual	Yes for adults, no for children
Right To Play, Pakistan					●		●	RCT	Yes for peer violence
Peace Education Afghanistan		●	●		●		●	Modified interrupted time series	Yes for peer violence
Sources of Strength, Kenya					●			RCT + qual	No

**Table 3:** Mapping of the 10 elements of design and implementation on to the different interventions.

	Essential Elements of Intervention Design				Essential Elements of Intervention Implementation		Elements of Intervention Design That Are Necessary Where Relevant to the Approach				Evaluation	Outcome
	Rigorously planned, robust ToC, attuned to the local context	Addressing multiple drivers of VAWG	Support for survivors	Working with men and women	Optimal intensity	Sufficient, well-selected, trained and supported staff and volunteers	Gender and social empowerment and positive interpersonal relations through group activities	Participatory learning methods, emphasising empowerment, critical reflection and communication skills	Carefully designed user-friendly manuals systematically followed	Age-appropriate design with time for learning and an engaging pedagogy	RCT, quasi-experimental study or modified interrupted time series/mixed methods	Evidence of VAWG or peer violence prevention
Interventions that showed evidence of impact in reducing VAWG												
Indashyikirwa couples, Rwanda	●	●	●	●	●	●	●	●	●		RCT	Yes
Stepping Stones Creating Futures South Africa	●	●	●	●	●	●	●	●	●		RCT	Yes for men, no for women
VATU, Zambia	●	●	●	●	●	●	❖		●		RCT	Yes for adults, no for children
Rural Response System, Ghana	●	●	●	●	●	●	❖				Quasi-experimental	Yes for women
Transforming Masculinities, DRC	●	●	●	●	●	●	●	●	●		Mixed methods	Yes
Zindagii Shoista, Tajikistan	●	●	●	●	●	●	●	●	●		Mixed methods	Yes
Sammanit Jeevan, Nepal	●	●	●	●	●	●	●	●	●		Mixed methods	Yes
Interventions showing no evidence of impact in reducing VAWG												
HERrespect Bangladesh	❖	●		❖		●	●	●	●		Quasi-experimental	No
Samvedana Plus, India	●	●	●	●	❖	●	●	●	●		RCT	No
Women’s Empowerment Programme, Afghanistan	❖	❖		❖	●	●	●		●		RCT	No
Indashyikirwa community, Rwanda	●	●	●	●	❖	❖	❖	❖			RCT	No
Change Starts at Home, Nepal	●	●		●	❖	❖	●	●	●		RCT	No
Sonke Change, South Africa	❖	❖		●	❖	❖	●	❖	❖		RCT	No
Interventions evaluated with children (with and without impact)												
Right To Play, Pakistan	●	●		●	●	●	●	●	●	●	RCT	Yes for peer violence
Peace Education Afghanistan	●	●		●	●	●	●		●	●	Modified interrupted time series	Yes for peer violence
Impower, Kenya				●	❖	●			❖		RCT	No
Sources of Strength, Kenya		❖		●	❖	●					RCT	No

## References

[1] (2021). United Nations Inter-Agency Working Group on Violence against Women Estimation and Data. Violence against Women Prevalence Estimates, 2018. Global, Regional and National Prevalence Estimates for Intimate Partner Violence against Women and Global and Regional Prevalence Estimates for Non-Partner Sexual Violence against Women.

[2] Abrahams N., Mhlongo S., Dunkle K., Chirwa E., Lombard C., Seedat S., Kengne A.P., Myers B., Peer N., Garcia-Moreno C. (2021). Increase in HIV incidence in women exposed to rape: A comparative cohort study. AIDS.

[3] Jewkes R.K., Dunkle K., Nduna M., Shai N. (2010). Intimate partner violence, relationship power inequity, and incidence of HIV infection in young women in South Africa: A cohort study. Lancet.

[4] Devries K.M., Child J., Bacchus L.J., Mak J., Falder G., Graham K., Watts C., Heise L. (2014). Intimate partner violence victimization and alcohol consumption in women: A systematic review and meta-analysis. Addiction.

[5] Devries K.M., Mak J.Y., Bacchus L.J., Child J., Falder G., Petzold M., Astbury J., Watts C.H. (2013). Intimate Partner Violence and Incident Depressive Symptoms and Suicide Attempts: A Systematic Review of Longitudinal Studies. PLoS Med..

[6] Potter L.C., Morris R., Hegarty K., García-Moreno C., Feder G. (2021). Categories and health impacts of intimate partner violence in the World Health Organization multi-country study on women’s health and domestic violence. Int. J. Epidemiol..

[7] Oram S., Khalifeh H., Howard L.M. (2017). Volence Against Women and Mental Health. Lancet Psychiatry.

[8] World Health Organisation (2013). Global and Regional Estimates of Violence against Women. Prevalence and Health Effects of Intimate Partner Violence and Non-Partner Sexual Violence.

[9] Ramsoomar L., Gibbs A., Chirwa E.D., Dunkle K., Jewkes R. (2021). Pooled analysis of the association between alcohol use and violence against women: Evidence from four violence prevention studies in Africa. BMJ Open.

[10] Kerr-Wilson A., Fraser E., Gibbs A., Ramsoomar L., Parke A., Maqbool H., Jewkes R. (2020). What Works to Prevent Violence Against Women and Girls? Evidence Review of Interventions to Prevent Violence against Women and Girls.

[11] Parcesepe A.M., L′engle K.L., Martin S.L., Green S., Sinkele W., Suchindran C., Speizer I.S., Mwarogo P., Kingola N. (2016). The impact of an alcohol harm reduction intervention on interpersonal violence and engagement in sex work among female sex workers in Mombasa, Kenya: Results from a randomized controlled trial. Drug Alcohol Depend..

[12] Jewkes R., Willan S., Heise L., Washington L., Shai N., Kerr-Wilson A., Christofides N. (2020). Effective Design and Implementation Elements in Interventions to Prevent Violence Against Women and Girls.

[13] Weber E.P., Belsky J.M., Lach D., Cheng A.S. (2014). The Value of Practice-Based Knowledge. Soc. Nat. Resour..

[14] Karmaliani R., McFarlane J., Khuwaja H.M.A., Somani Y., Bhamani S.S., Ali T.S., Asad N., Chirwa E.D., Jewkes R. (2020). Right to Play’s intervention to reduce peer violence among children in public schools in Pakistan: A cluster-randomized controlled trial. Glob. Health Action.

[15] Corboz J., Siddiq W., Hemat O., Chirwa E.D., Jewkes R. (2019). What Works to Prevent Violence Against Children in Afghanistan? Findings of an interrupted time series evaluation of a school-based peace education and community social norms change intervention in Afghanistan. PLoS ONE.

[16] Gibbs A., Corboz J., Chirwa E., Mann C., Karim F., Mohammed S., Mecagni A., Maxwell-Jones C., Noble E., Jewkes R. (2020). Evaluating the impacts of a combined social and economic empowerment training programme on intimate partner violence, gender norms, and livelihoods amongst women in Afghanistan: An individually randomized control trial and qualitative study. BMJ Glob. Health.

[17] Naved R., Al Mamun M., Parvin K., Willan S., Gibbs A., Jewkes R. (2021). Learnings from the evaluation of HERrespect: A factory-basedn intervention to prevent intimate partner and workplace violence against female garment workers in Bangladesh. Glob. Health Action.

[18] Naved R., Rahman T., Willan S., Jewkes R., Gibbs A. (2018). Female garment workers’ experiences of violence in their homes and workplaces in Bangladesh: A qualitative study. Soc. Sci. Med..

[19] Jewkes R., Flood M., Lang J. (2015). From work with men and boys to changes of social norms and reduction of inequities in gender relations: A conceptual shift in prevention of violence against women and girls. Lancet.

[20] Ellsberg M., Arango D.J., Morton M., Gennari F., Kiplesund S., Contreras M., Watts C. (2015). Prevention of violence against women and girls: What does the evidence say?. Lancet.

[21] Gibbs A., Dunkle K., Ramsoomar L., Willan S., Jama Shai N., Chatterji S., Naved R., Jewkes R. (2020). New learnings on drivers of men’s physical and/or sexual violence against their female partners, and women’s experiences of this, and the implications for prevention interventions. Glob. Health Action.

[22] Jewkes R., Nduna M., Levin J., Jama N., Dunkle K., Puren A., Duvvury N. (2008). Impact of Stepping Stones on incidence of HIV, HSV-2 and sexual behaviour in rural South Africa: Cluster randomised controlled trial. Br. Med J..

[23] Dunkle K., Stern E., Chatterji S., Heise L. (2020). Effective prevention of intimate partner violence through couples training: A randomised controlled trial of Indashyikirwa in Rwanda. BMJ Glob. Health.

[24] Christofides N., Hatcher A., Rebombo D., McBride R., Munshi S., Pino A., Abdelatif N., Levin J., Jewkes R. (2020). Effectiveness of a multi-level intervention to reduce men’s perpetration of intimate partner violence: A cluster randomised controlled trial. Trials.

[25] Hatcher A.M., McBride R.-S., Rebombo D., Munshi S., Khumalo M., Christofides N. (2020). Process evaluation of a community mobilization intervention for preventing men’s partner violence use in peri-urban South Africa. Eval. Program Plan..

[26] Murray L., Kane J., Glass N., van Wyk S., Melendez F., Paul R., Danielson C.K., Murray S., Mayeya J., Simenda F. (2020). Effectiveness of the Common Elements Treatment Approach (CETA) in reducing intimate partner violence and hazardous alcohol use in Zambia (VATU): A randomised controlled trial. PLoS Med..

[27] Stern E., Carlson K. (2019). Indashyikirwa safe spaces: Response mechanisms for survivors of IPV within a Rwandan prevention programme. Soc. Sci..

[28] Ogum Alangea D., Addo-Lartey A.A., Chirwa E.D., Sikweyiya Y., Coker-Appiah D., Jewkes R., Adanu R.M.K. (2020). Evaluation of the rural response system intervention to prevent violence against women: Findings from a community-randomised controlled trial in the Central Region of Ghana. Glob. Health Action.

[29] Mastonshoeva S., Shonasimova S., Gulyamova P., Jewkes R., Shai N., Chirwa E., Myrttinen H. (2019). Mixed Methods Evaluation of Zindagii Shoista (Living with Dignity) Intervention to Prevent Violence Against Women in Tajikistan.

[30] Shai N., Pradhan G.D., Shrestha R., Adhikari A., Chirwa E., Kerr-Wilson A., Jewkes R. (2020). “I got courage from knowing that even a daughter-in-law can earn her living”: Mixed methods evaluation of a family-centred intervention to prevent violence against women and girls in Nepal. PLoS ONE.

[31] Javalkar P., Platt L., Prakash R., Beattie T.S., Collumbien M., Gafos M., Ramanaik S., Davey C., Jewkes R., Watts C. (2019). Effectiveness of a multilevel intervention to reduce violence and increase condom use in intimate partnerships among female sex workers: Cluster randomised controlled trial in Karnataka, India. BMJ Glob. Health.

[32] Clark C., Shrestha B., Gerguson G., Shrestha P., Calvert C., Gupta J., Batayeh B., Bergenfeld I., Oakes M. (2019). Impact of the Change Starts at Home Trial on Women’s Experience of IPV in Nepal. Soc. Sci. Med. Popul. Health.

[33] Rosenman E., Sarnquist C., Friedberg R., Amuyunzu-Nyamongo M., Oguda G., Otieno D., Baiocchi M. (2020). Empirical Insights for Improving Sexual Assault Prevention: Evidence From Baseline Data for a Cluster-Randomized Trial of IMPower and Sources of Strength. Violence Against Women.

[34] Wingood G.M., DiClemente R.J. (2000). Application of the Theory of Gender and Power to Examine HIV-Related Exposures, Risk Factors, and Effective Interventions for Women. Health Educ. Behav..

[35] Lee J. (2001). The Empowerment Approach to Social Work Practice.

[36] Campbell C., Jovchelovitch S. (2000). Health, community and development: Towards a social psychology of participation. J. Community Appl. Psychol..

[37] Friere P. (1993). Pedagogy of the Oppressed.

[38] Pretty J.N., Guijt I., Thompson J., Scoones I., Faul-Doyle R. (1995). A Trainer’s Guide to Participatory Learning and Action.

[39] Stern E., Willan S., Gibbs A., Myrttinen H., Washington L., Sikweyiya Y., Addo-Lartey A., Mastonshoeva S., Jewkes R. (2020). Pathways of change: Qualitative evaluations of intimate partner violence prevention programmes in Ghana, Rwanda, South Africa and Tajikistan. Cult. Health Sex..

[40] Jewkes R., Wood K., Duvvury N. (2010). I woke up after I joined Stepping Stones’: Meanings of an HIV behavioural intervention in rural South African young people’s lives. Health Educ. Res..

[41] Scott C. (2015). Futures of Learning 3: What kind of pedagogies for the 21st Century?. Educational Research and Foresight Working Papers No15.

[42] Jewkes R., Morrell R., Hearn J., Lundqvist E., Blackbeard D., Lindegger G., Quayle M., Sikweyiya Y., Gottzen L. (2015). Hegemonic masculinity: Combining theory and practice in gender interventions. Cult. Health Sex..

[43] Gevers A., Taylor K., Droste M., Williams J. (2019). Lessons Learned about Promary Prevention of Violence against Women and Girls in Asia and the Pacific Region.

[44] Starmann E., Heise L., Kyegombe N., Devries K., Abramsky T., Michau L., Musuya T., Watts C., Collumbien M. (2018). Examining diffusion to understand the how of SASA!, a violence against women and HIV prevention intervention in Uganda. BMC Public Health.

